# 3,6-Dimethoxythieno[3,2-b]thiophene-Based Bifunctional Electrodes for High-Performance Electrochromic Supercapacitors Prepared by One-Step Electrodeposition

**DOI:** 10.3390/polym16162313

**Published:** 2024-08-15

**Authors:** Zhixuan Yu, Rui Wang, Huayu Tang, Ding Zheng, Junsheng Yu

**Affiliations:** State Key Laboratory of Electronic Thin Films and Integrated Devices, School of Optoelectronic Science and Engineering, University of Electronic Science and Technology of China, Chengdu 610054, China; 202221050502@std.uestc.edu.cn (Z.Y.); 18738678210@163.com (R.W.); t17856692936@163.com (H.T.)

**Keywords:** one-step electrodeposition, 3,6-dimethoxythieno[3,2-b]thiophene, electrochromism, controlled color change, supercapacitor

## Abstract

An integrated visual energy system consisting of conjugated polymer electrodes is promising for combining electrochromism with energy storage. In this work, we obtained copolymer bifunctional electrodes poly(3,6-dimethoxythieno[3,2-b]thiophene-*co*-2,3-dihydrothieno[3,4-b][1,4]dioxin-3-ylmethanol)(P(TT-OMe-*co*-EDTM)) by one-step electrochemical copolymerization, which exhibits favorable electrochromic and capacitive energy storage properties. Because of the synergistic effect of PTT-OMe and PEDTM, the prepared copolymers show better flexibility. Moreover, the morphology and electrochemical properties of the copolymers could be adjusted by depositing different molar ratios of 3,6-dimethoxythieno[3,2-b]thiophene (TT-OMe) and 2,3-dihydrothieno[3,4-b][1,4] dioxin-3-ylmethanol (EDTM). The P(TT-OMe-*co*-EDTM) electrodes realized a high specific capacitance (190 F/g at 5 mV/s) and recognizable color conversion. This work provides a novel and simple way to synergistically improve electrochromic and energy storage properties and develop thiophene-based conducting polymers for electrochromic energy storage devices.

## 1. Introduction

As the demand for renewable energy rises, the development of sustainable energy storage devices has become a popular research direction [1]. Supercapacitors (SCs), characterized by a long cycle life, a high power density, and fast charge/discharge rates [2,3,4,5,6], stand out as the most promising candidate among the energy storage devices for renewable energy systems. Among others, electrochromic supercapacitors (ESCs) have the capability of reversible color transformation in specific electrode materials during the electrochemical oxidation/reduction processes induced by an applied voltage [7]. The capacity of ESCs to both change colors and store energy enables them to fulfill the essential requirements for electronic devices, smart wearables, and military gear, which has emerged as a hot area of research [8,9,10,11]. However, the exploitation of bifunctional electrode materials for electrochromic supercapacitors is particularly critical for next-generation energy storage devices.

Over the years, the bifunctional electrode materials for ESCs have consisted of two main categories: transition metal oxides [12,13,14] and conducting polymers [15,16,17]. Transition metal oxides, such as MnO_2_ and RuO_2_, have emerged as the most utilized substances. Their prevalent use is attributed to their commendable performance, such as environmental friendliness and high energy density. However, transition metal oxide materials suffer from drawbacks, such as low conductivity, extended response times, limited flexibility, and single color variability [18,19,20]. In contrast to transition metal oxides, conductive polymers, such as PANI, PPy, PTh, and PEDOT, which offer advantages like diverse color options, rapid response times, and exceptional flexibility, herald a promising trend for future large-scale device manufacturing in industry and application as electrode materials for flexible functional devices [21,22,23]. Furthermore, the properties of these conductive polymers could be easily adjusted through various physical and chemical strategies, enhancing their suitability for multiple applications [24,25,26,27,28].

Among the different conducting polymers, Polythiophene (PTh) and its analogue Poly2,3-dihydrothieno[3,4-b][1,4]dioxin-3-ylmethanol (PEDTM) are extensively researched due to their ease of synthesis, flexibility, and excellent electrochemical properties [29,30]. Moreover, the derivatives of thiophene-based polymers, exemplified by fused thiophenes (e.g., thieno[3,2-b]thiophene, TT), have attracted extensive attention for their extended π-conjugated structures, which can significantly increase the charge carrier mobility [31]. Furthermore, the modification of the side chains can further refine the morphology of conductive polymer films, which is beneficial for enhancing their electrochemical performance [32]. For instance, TT modified with methoxy side chains demonstrates exceptional electropolymerization capability, and its polymer present remarkable electrochromic characteristics, positioning it as a promising material in the field of advanced organic electronics. Nonetheless, poly(3,6-dimethoxythieno[3,2-b]thiophene) (TT-OMe)P(TT-OMe) polymers directly electropolymerized from their monomers exhibit poor electrochemical activity due to an unsatisfactory microstructure, constraining their use in electrochemical energy storage devices [33,34,35]. Electrochemical copolymerization offers the advantage of precisely tailoring the microstructure of conducting polymer films through a controlled synthesis process directly on the electrode surfaces, which provides a promising platform for developing high-performance electrochemical devices.

In this study, P(TT-OMe-*co*-EDTM) copolymer thin films were synthesized by one-step electrochemical polymerization. By adjusting the molar ratio of TT-OMe and EDTM, bifunctional electrodes with a customizable morphology and flexibility were achieved. These electrodes exhibit an enhanced specific capacitance (from 104 F/g to 190 F/g) and controllable color conversion (from purple to yellow). A supercapacitor is a novel energy storage device with the features of a high power density, fast charging and discharging speeds, high reliability, environmental friendliness, and so on. Furthermore, when integrated into an electrochromic supercapacitor, the charge status can be visually monitored due to the reversible and stable chromatic changes. This suggests the potential for developing P(TT-OMe-co-EDTM) electrodes into smart electrochromic supercapacitors.

## 2. Experiment

### 2.1. Materials

3,6-dimethoxythieno[3,2-b]thiophene(TT-OMe) was purchased from Bide Pharmatech Ltd., Shanghai, China, and 2,3-dihydrothieno[3,4-b][1,4]dioxin-3-ylmethanol (EDTM) was purchased from Innokai Technology Ltd., Beijing, China. Tetrabutylammonium hexafluorophosphate (Bu_4_NPF_6_, 99%; Energy Chemical, Shanghai, China) was from Bide Pharmatech Ltd. and dried under a vacuum at 80 °C for 24 h before use. Aceto-nitrile (ACN, AR, Beijing, China) was purchased from Beijing InnoChem Science & Technology Co., Ltd., Beijing, China.

### 2.2. Measurement and Characterization

The CHI 660E electrochemical workstation (Shanghai Chenhua Ins., Shanghai, China) was used in all electrochemical measurements. The electrochemical polymerization of the monomers was performed in a precisely technical standard-setting three-electrode system using Pt wire as the counter electrode, Ag/AgCl wire as the reference electrode, and ITO-coated glass as the working electrode to supply a superior polymerization stability and deposition on the surface area (effective area = 1.5 cm^2^). The 3600i Plus UV-Vis-NIR spectrophotometer (Tokyo, Japan) was used to evaluate the absorption spectra. We used the ZEISS-Sigma-300 scanning electron microscope (SEM) (Oberkochen, Germany) to measure the morphology of samples. Infrared spectra were obtained by using the Nicolet-iS10 FTIR spectrometer with KBr pellets (Thermo Fisher Scientific, Waltham, MA, USA). The thermal property was analyzed by thermogravimetric analysis (TGA) on an NETZSCH STA 449F3 (NETZSCH-Gerätebau GmbH, Selb, Germany) in the temperature range of 200–1000 °C with a heating rate of 10 °C/min under N_2_ flow. The bending stability of the polymer film was assessed at a certain bending angle and under the same mechanical stress.

### 2.3. Fabricating the Polymer Electrodes

**Figure 1:**
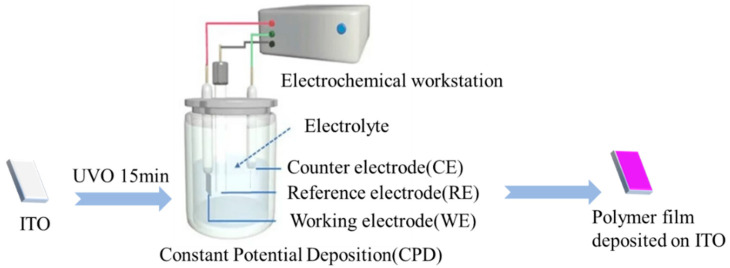
Preparation process of polymer film (P(TT-OMe-*co*-EDTM), PTT-OMe, PEDTM).

The polymer film was electrodeposited on the surface of ITO-coated glass by a constant potential method in a standard-setting three-electrode system, as shown in Figure 1. The effective area (1.5 cm^2^) of the working electrode was immersed in 0.1 M Bu_4_NPF_6_/ACN solution, including 0.05 M monomer or a mixture of TT-OMe and EDTM monomers in molar ratios of 10:1, 8:1, and 4:1. The polymer films were deposited with 1.4 V constant electrical potential (amount of deposition = 5 mC/cm^2^) on the ITO for UV-Vis spectroscopy measurement. The films were electrodeposited using the same method for 3500 s, and then immersed the films adhered to the ITO-coated glass substrates in deionized water, peeled off, and dried to yield free-standing and soft films. For TGA and FTIR testing, the polymers were electrodeposited on ITO (2 cm × 1 cm), and then scraped off the ITO surface, and then cleaned.

The monomers were electrochemically polymerized potentiostatically at 1.4 V. Assuming a current efficiency (η) of 100%, the mass of polymers was computed following Equation (1) below [36].

(1) $$m=\frac{\left(nQ_{dep}\right)\left(M\right)}{FZ}$$

where *M* is the molecular weight of the monomer. *F* is the Faraday constant (96,485 C/mol). *Z* is the quantity of electrons transferred per monomer attached to the polymer, and *Z* = 2 + *f*. The part of the charge *f* is known as the doping level, with *f* being approximately 0.33 [37].

All electrochemical characterizations of the polymer electrodes were carried out using the standard-setting three-electrode system with 0.1 M Bu_4_NPF_6_/ACN electrolyte solution. For electrochemical characterization, polymer-coated ITO was used as the working electrode, Pt wire was used as the counter electrode, and Ag/AgCl was used as the reference electrode. Cyclic voltammetry (CV) curves were used to calculate the specific capacitance of the polymers electrode using Equation (2) [38]:

(2) $$C_{s}=\frac{\int_{E1}^{E2}i\left(E\right)d\left(E\right)}{2mv\Delta V}\;\;\;$$

where $\int_{E1}^{E2}i\left(E\right)d\left(E\right)$ is the total integrated charge through the anode and cathode scans in the CV curve, m is the mass of the active polymer deposited on the electrode, v is the scan rate, ΔV is the potential window for CV, and $C_{s}$ is the specific capacitance.

In addition, the specific capacitance can be calculated from the CD curves using Equation (3) [39,40]:

(3) $$C=\frac{I\times \Delta T_{d}}{m\times \Delta V}$$

where *I* and $\Delta T_{d}$ are the discharge current and the discharge time, respectively, *m* is the mass of the polymer film electrode, and ΔV is the potential window in the discharging process.

## 3. Results and Discussion

### 3.1. Electrochemical Polymerization of Monomers

**Figure 2:**
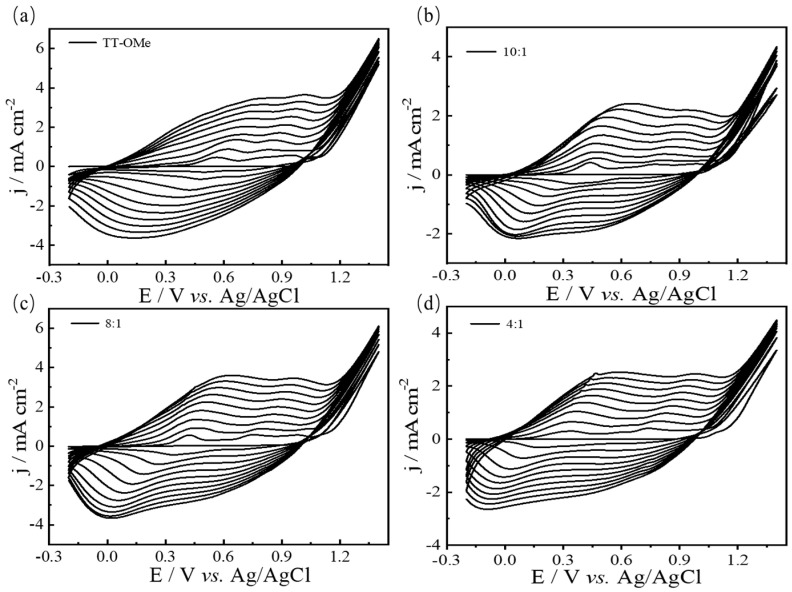
CVs of 0.05 M TT−OMe (**a**), and EDTM monomer mixtures with different molar ratios of 10:1 (**b**), 8:1 (**c**), and 4:1 (**d**) in ACN-Bu_4_NPF_6_ at 50 mV/s.

The electrochemical polymerization of the monomer and the mixture occurred in 0.1 M ACN-Bu_4_NPF_6_, as depicted in Figure 2 and Figure S1. The appearance of a redox peak in the second cycle indicates the formation of a conducting polymer in the initial cycle [41]. The redox peak current density gradually increases as the cycle grows, indicating that the amount of polymer on the working electrode is increasing. At the same time, the potential of the redox peaks has shifted because of the higher resistance of the conducting polymers grown on the working electrode [42]. In addition, with the increase in EDTM content in the electrochemical polymerization process, the reduction peak gradually moved to a negative potential, which suggested that the copolymer of TT-OMe and EDTM was formed. It is generally believed that there are two mechanisms for the electro-polymerization of heterocyclic compounds, either radical–radical or radical–monomer coupling [43,44]. The monomer undergoes oxidation to form a cationic radical, which then reacts with the neutral molecules to produce a cationic dimer. This dimer loses electrons to become a neutral dimer, and the process continues through oxidation, recombination, and deionization, ultimately yielding the final polymer product [45]. In addition, the CV behavior of the copolymers is quite different from the CV of the monomers, which results in the enhanced electrochemical properties of the copolymers [45].

### 3.2. Morphology of Polymers

**Figure 3:**
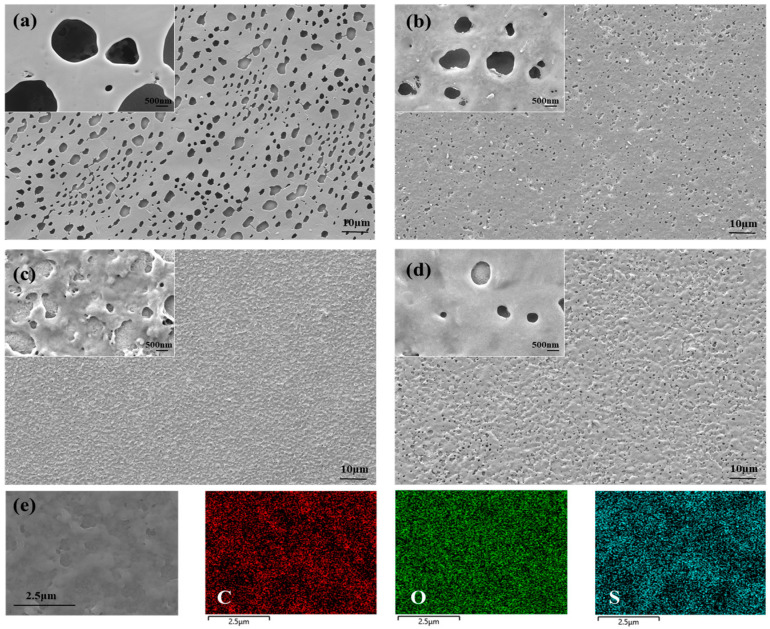
SEM images of (**a**) PEDTM, (**b**) P_10:1_(TT-OMe-*co*-EDTM), (**c**) P_8:1_(TT-OMe-*co*-EDTM), and (**d**) P_4:1_(TT-OMe-*co*-EDTM)-coated ITO glass, and (**e**) EDS elemental color mapping images of P_8:1_(TT-OMe-*co*-EDTM).

In order to compare the morphologies of the polymers PTT-OMe, PEDTM, P_10:1_(TT-OMe-*co*-EDTM), P_8:1_(TT-OMe-*co*-EDTM), and P_4:1_(TT-OMe-*co*-EDTM), the surface morphology was recorded by using SEM techniques, and the material elements were analyzed by Energy-Dispersive X-ray Spectroscopy (EDS). And the combination of the two monomers is beneficial in the morphology modulation of polymers. Figure 3a–d shows the low- and high-resolution morphologies of PTT-OMe, PEDTM, P_10:1_(TT-OMe-*co*-EDTM), P_8:1_(TT-OMe-*co*-EDTM), and P_4:1_(TT-OMe-*co*-EDTM), respectively. The SEM images reveal that three copolymer images are porous and evenly distributed over the entire surface to form a film, a critical feature that promotes charge storage and ion diffusion as well as reduces the dead volume as it allows for the maximum penetration of electrolyte ions [46]. As shown in Figure S2 and Figure 3a, the PEDTM film has a relatively large number of round holes on its surface, whereas the PTT-OMe film has a dense surface. This explains why the film of PEDTM is a flexible free-standing film. As the proportion of EDTM in the precursor mixture increases from one eleventh to one fifth, the polymer surface gradually forms more pores, with reduced pore depth and increased surface roughness. It is worth noting that at a mixing ratio of 8:1, the surface becomes compact, and the microporous layers are graded. This copolymer-modified nanostructure creates an effective diffusion path that will facilitate ion transport, greatly accelerating ion penetration on the surface of an electrode and increasing the effective use of the electrode material [46]. The morphological changes in the P(TT-OMe-*co*-EDTM) films are attributed to the synergistic effect of TT-OMe and EDTM. As can be seen from the CV of monomer polymerization, the EDTM (Figure S1) monomer polymerizes more slowly than TT-OMe, resulting in a porous and smooth film after polymerization at the same driving voltage. The different ratios of TT-OMe to EDTM monomers during the copolymerization process can affect the polymerization behavior, leading to morphological changes of polymers, and resulting in copolymer modification. The elemental mapping images of P_8:1_(TT-OMe-*co*-EDTM) (Figure 3e) confirm the presence of carbon (C), oxygen (O), and sulfur (S) as the major components present with atomic percentages of 49.96%, 47.57%, and 2.48%, respectively (Table S1).

### 3.3. Structure, Thermal Analysis, and Mechanical Bending of Polymers

**Figure 4:**
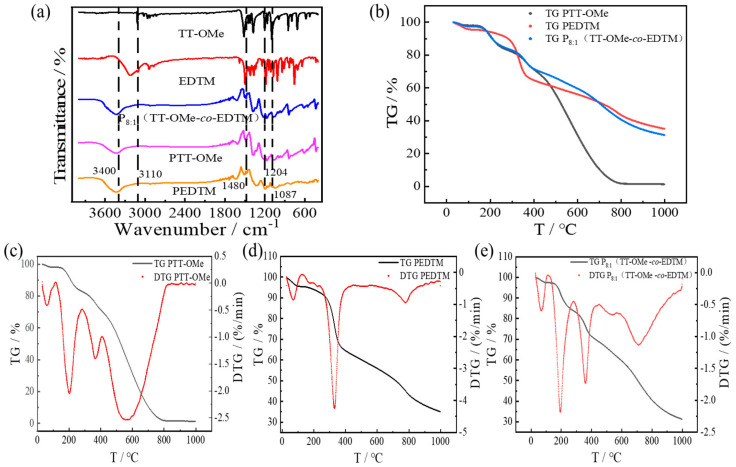
(**a**) FTIR spectra of TT−OMe, EDTM, P_8:1_(TT−OMe-*co*-EDTM), PTT−OMe, and PEDTM; (**b**) TG curves of PTT−OMe, PEDTM, and P_8:1_(TT−OMe-*co*-EDTM); (**c**) TG and DTG curves of PTT−OMe; (**d**) TG and DTG curves of PEDTM; and (**e**) TG and DTG curves of P_8:1_(TT−OMe-*co*-EDTM).

Figure 4a shows the FT-IR spectra of the monomers and polymers. It can be seen from the figure that the intensity of the peaks at around 1204 cm^−1^ and 1087 cm^−1^ (represented of C-O-C bonding) [47] gradually increases as the copolymerization of the two monomers is completed. For the FTIR spectrum of P_8:1_(TT-OMe-*co*-EDTM), the characteristic peak of it at 3400 cm^−1^ corresponds to the stretching of the –OH bond. In addition, EDTM and TT-OMe show a peak of aromatic C=C-H (3110 cm^−1^), but P_8:1_(TT-OMe-*co*-EDTM) did not show this. Moreover, EDTM, PEDTM, PTT-Ome, and P_8:1_(TT-OMe-*co*-EDTM) show a peak of methylene C-C-H (1480 cm^−1^), except for TT-OMe [14]. For the FTIR spectra of P_8:1_(TT-OMe-*co*-EDTM), the characteristic peaks of PEDTM and PTT-OMe all appear in the composite films of P_8:1_(TT-OMe-*co*-EDTM). Through the above analysis, we can conclude that the monomer blends of EDTM and TT-OMe were successfully prepared as copolymers by electrochemical polymerization. In the field of supercapacitors, the stability of conducting polymers is very significant. The thermal stability analysis of the polymer films was carried out in a nitrogen atmosphere at a temperature increase rate of 10 °C/min up to 1000 °C, as shown in Figure 4b. At 140 °C and below, the weight loss of PTT-OMe, PEDTM, and P_8:1_(TT-OMe-*co*-EDTM) was about 2.07%, 4.63%, and 2.69%, respectively, which may be related to the evaporation of water or the removal of other impurities. From 140 °C to 450 °C, the weight loss of PTT-OMe, PEDTM, and P_8:1_(TT-OMe-*co*-EDTM) mainly originated from the release flow of oligomers and the decomposition of dopant ions. With further increase in temperature, the weight loss was mainly related to the degradation of the polymer backbone and the skeleton. The synergistic effect of the mixing of TT-OMe and EDTM monomers at the molecular level during copolymerization resulted in the copolymer mass retention reaching 50% at 712 °C. In addition, the temperatures at the maximum decomposition rates of PTT-OMe, PEDTM, and P_8:1_(TT-OMe-*co*-EDTM) were 575 °C, 330 °C, and 194 °C, respectively. The test results confirm that the thermal stability of the P_8:1_(TT-OMe-*co*-EDTM) composite copolymer has been substantially improved compared with that of the PTT-OMe single polymer. 

**Figure 5:**
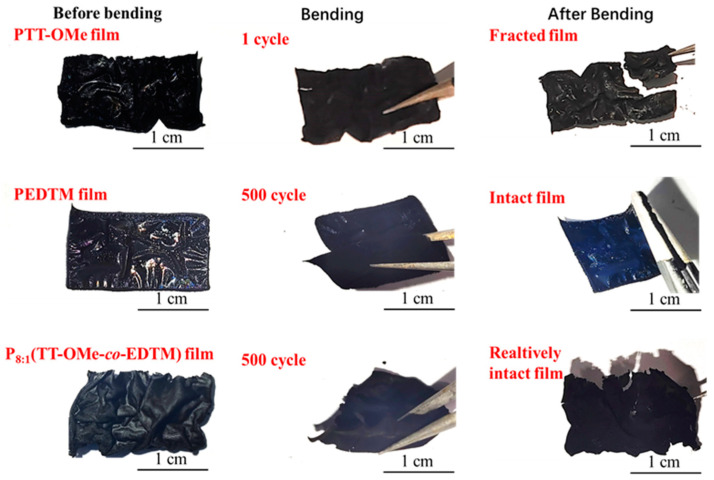
Photographs of the free-standing films, and the bending test of PTT-OMe, PEDTM, and P_8:1_(TT-OMe-*co*-EDTM).

Additionally, the mechanical bending cycle test of the as-prepared conducting polymer films was conducted, as depicted in Figure 5. The PEDTM film exhibited exceptional robustness, remaining intact without any significant degradation even after 500 bending cycles. In contrast, the PTT-OMe film demonstrated fragility, being prone to fracturing and lacking bendability. This observation corroborates the notion that the flexibility of copolymer films is predominantly imparted by the incorporation of the PEDTM moiety, resulting in copolymer films that retain their structural integrity after 500 mechanical bending cycles, thereby showcasing superior mechanical flexibility. The enhanced flexibility of the copolymer films can be ascribed to alterations in the conjugated backbone structure of the EDTM and TT-OMe monomers subsequent to the electrochemical deposition process. Furthermore, improved mechanical bending stability will facilitate the integration of these films into flexible electronic devices, enhancing their reliability and longevity for practical applications in flexible electronics.

### 3.4. Electrochemical Performance of Polymer Electrodes

**Figure 6:**
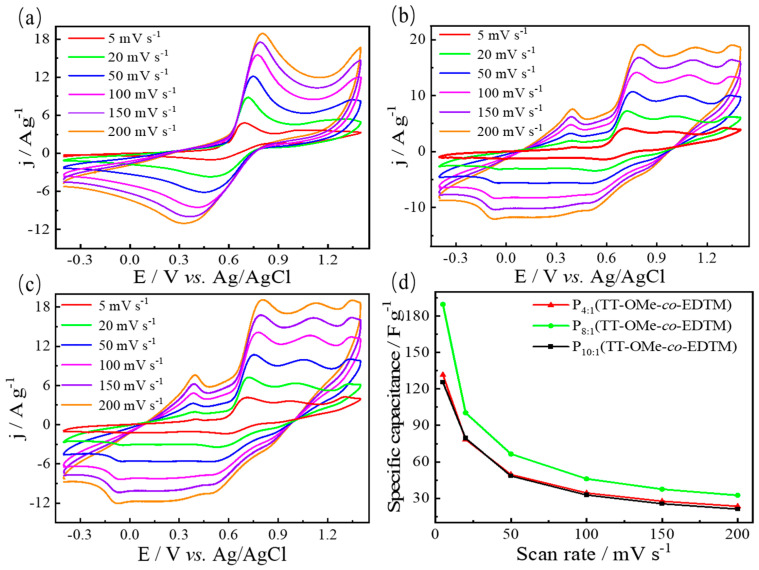
CV curves of 0.05 M (**a**) P_10:1_(TT−OMe-*co*-EDTM), (**b**) P_8:1_(TT−OMe-*co*-EDTM), (**c**) and P_4:1_(TT−OMe-*co*-EDTM) in ACN−Bu_4_NPF_6_ at different scan rates. (**d**) Plots of specific capacitances versus scan rates of polymers.

The CV curves for P_10:1_(TT-OMe-*co*-EDTM), P_8:1_(TT-OMe-*co*-EDTM), and P_4:1_(TT-OMe-*co*-EDTM), PTT-OMe, and PEDTM are presented in Figure 6 and Figure S3, respectively. The CV measurements of the polymer thin films were conducted in 0.1 M ACN-Bu_4_NPF_6_ electrolyte within a potential window from −0.4 to 1.4 V at various scan rates (5, 20, 50, 100, 150, and 200 mV/s), enabling a detailed investigation of their capacitive properties. As depicted in Figure 6, the conducting polymer films maintained relatively distinct redox peaks even as the scan rate increased, indicating that the electrode materials underwent stable and efficient electrochemical redox processes, which are crucial for their application in high-performance energy storage devices. Furthermore, the CV curve shape remained largely unchanged at the higher scan rates, signifying the material has a superior pseudocapacitive performance. The three copolymer films exhibited reversible redox reaction behaviors across a broad potential window (1.8 V), indicative of their excellent electrochemical activity [48]. Figure S3 shows that the conducting polymer films derived from the EDTM monomer exhibited higher current densities compared to those formed from the TT-OMe monomer via electropolymerization, suggesting a greater number of electroactive sites and a higher degree of electronic conductivity within the PEDTM-based films. The current densities of the copolymer films were found to be intermediate between those of the two homopolymer films (PTT-OMe and PEDTM), which indirectly confirms the successful combination of the two monomers.

Figure 6d reveals that the specific capacitances derived from the CV curves are 125 F/g, 190 F/g, and 132 F/g at a scan rate of 5 mV/s for P_10:1_(TT-OMe-*co*-EDTM), P_8:1_(TT-OMe-*co*-EDTM), and P_4:1_(TT-OMe-*co*-EDTM), respectively, outperforming PTT-OMe (104 F/g) and PEDTM (119 F/g), as indicated in Figure S3c. The suboptimal capacitive performance of the PTT-OMe thin film electrodes can be ascribed to their overly compact surface microstructure, which leads to a reduced electrochemically active surface area and a diminished rate of electrochemical reactions. The introduction of the EDTM monomer into the TT-OMe monomer, leveraging the porous microstructure of PEDTM (Figure 3a), fosters effective synergy between EDTM and TT-OMe, resulting in an increased electrochemically active surface area and an enhanced specific capacitance. Through a series of experiments exploring various monomer copolymerization ratios, the polymer films exhibited the most favorable electrochemical energy storage properties when the molar ratio of TT-OMe to EDTM monomers was 8:1. Moreover, the capacitive performance of the copolymer films prepared via an electrochemical copolymerization strategy significantly surpasses that of most previously reported bifunctional electrode materials (Table S2 [14,15,16,42,49,50,51,52,53,54,55]), highlighting the prominent advantages of these materials in the field of supercapacitor technology. Electrochemical stability can be measured by calculating the capacitance retention from a voltammogram of the first versus the last cycle. The electrochemical stability of PTT-OMe, PEDTM, and P(TT-OMe-*co*-EDTM) was calculated using repetitive CV data for 3000 cycles at 200 mV/s (Figure S4). The stability of PTT-OMe, PEDTM, and P(TT-OMe-*co*-EDTM) was 7.78%, 91.25%, and 39.15% after 500 cycles, respectively. In the future, we will choose specific dopants, improve the polymer preparation process, or design specific molecular structures to enhance the stability of conducting polymers.

**Figure 7:**
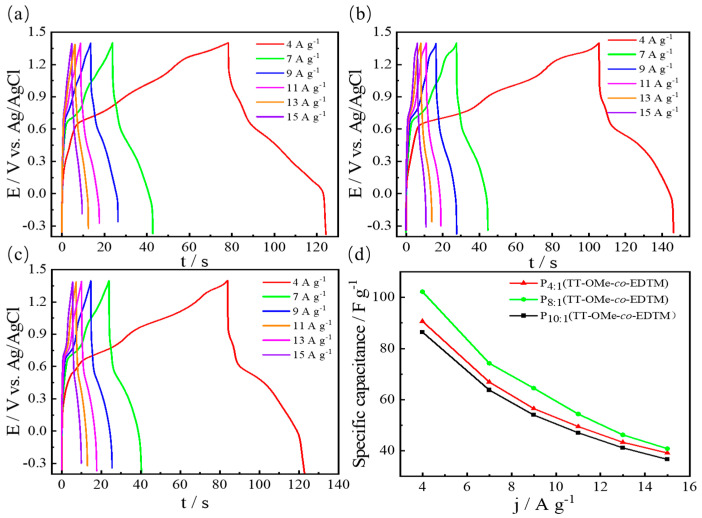
CD curves of 0.05 M (**a**) P_10:1_(TT−OMe-*co*-EDTM), (**b**) P_8:1_(TT−OMe-*co*-EDTM), and (**c**) P_4:1_(TT−OMe-*co*-EDTM) in ACN−Bu_4_NPF_6_ at different current densities. (**d**) Plots of specific capacitances versus current densities of polymers.

The galvanostatic charge–discharge (GCD) curves of the copolymers with different monomer ratios measured at different current densities are shown in Figure 7a–c. A nonlinear voltage–time curve shows the typical pseudocapacitance contribution of a polymer [56]. It can be found that the GCD curve changes drastically near 0.6–0.7 V, which indicates that the specific capacitance performance is mainly pseudocapacitive and is consistent with the CV curves [57]. Capacitance variation versus the current density is shown in Figure 7d. Increasing the current density will decrease the specific capacitance due to an insufficient redox reaction. According to the calculation formula of specific capacitance, the calculated specific capacitances are 65 F/g, 86 F/g, 102 F/g, and 91 F/g at 4 A/g for PTT-OMe, P_10:1_(TT-OMe-*co*-EDTM), P_8:1_(TT-OMe-*co*-EDTM), and P_4:1_(TT-OMe-*co*-EDTM), respectively. Specifically, as the current density increases, there is little interaction between the active sites on the electrode and the electrolyte ions [18]. Therefore, at high current densities, this ion diffusion limitation leads to a decrease in specific capacitance [18]. For the PTT-OMe electrode (Figure S5a), the charging and discharging times are very short when the current density is high, which indicates its poor capacitive performance. When the current density was increased from 4 to 15 A/g, the copolymer film electrode exhibited a better rate capability compared to that of the PTT-OMe electrode, with a capacitance retention of 27%. An increase in specific capacitance and an enhanced rate capability can be attributed to the introduction of porous PEDTM units in the PTT-OMe backbone, and the effective synergy of the two monomers ensures a higher specific surface area and efficient charge transportation.

**Figure 8:**
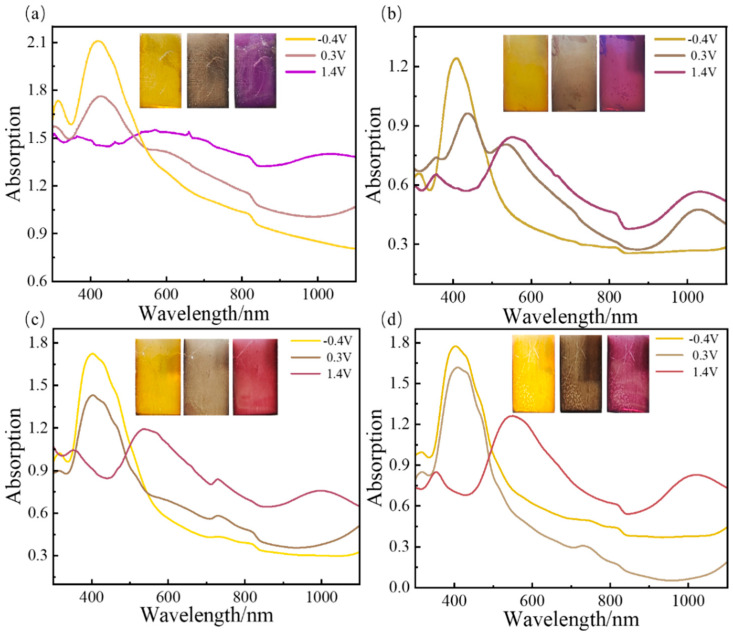
Absorption spectra of (**a**)TT−OMe, (**b**) P_10:1_(TT−OMe-*co*-EDTM), (**c**) P_8:1_(TT−OMe-*co*-EDTM), and (**d**) P_4:1_(TT−OMe-*co*-EDTM) films under different potentials in ACN−Bu_4_NPF_6_ electrolyte.

The absorption spectra of the five kinds of conducting polymer film are shown in Figure 8 and Figure S6. The PTT-OMe film displays light yellow coloration (indicative of a reduced state) at low potentials on the indium tin oxide (ITO) substrate. With the elevation of the potential, the film undergoes a transition to a purple hue (corresponding to an oxidized state). The maximum absorption peaks shift towards the blue region with an increasing EDTM content, with the peaks situated at 420 nm for PTT-OMe, 409 nm for P_10:1_(TT-OMe-*co*-EDTM), 404 nm for P_8:1_(TT-OMe-*co*-EDTM), 402 nm for P_4:1_(TT-OMe-*co*-EDTM), and 360 nm for PEDTM. The observed shift in the absorption peaks also indirectly confirms the transformation of the intrinsic π-conjugated structure of the conducting polymer during the electrochemical copolymerization process. As the applied voltage progresses (from −0.4 V to 0.3 V to 1.4 V), the ultraviolet absorption peaks attenuate, while new absorption bands emerge in the near-infrared spectrum, indicative of the formation of bipolar electrons and polarizers [49,58]. These copolymer films display pronounced color variations and rapid, reversible color transitions across a broad voltage spectrum. The observed color-shifting behavior in these polymer films promises to accelerate the development of electrochromic supercapacitors, which are useful in diverse applications, such as wearable electronics, energy-efficient windows, and smart sensors for building integration.

**Figure 9:**
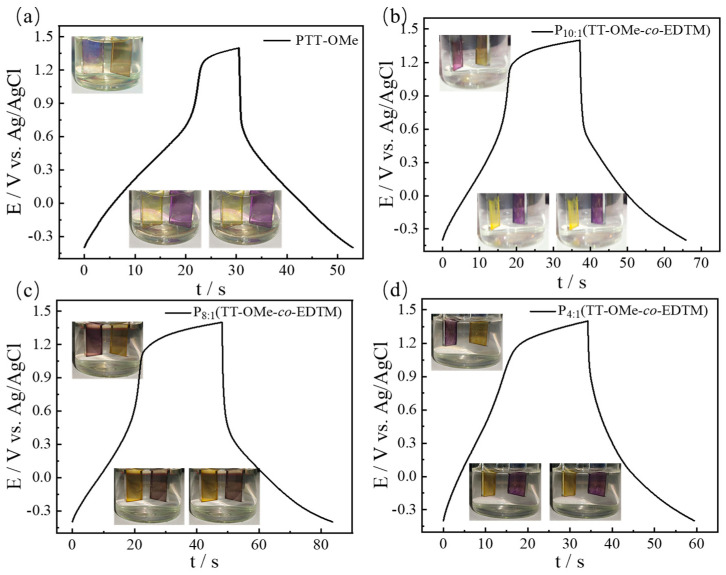
GCD curves and corresponding color change in (**a**) PTT−OMe, (**b**) P_10:1_(TT−OMe-*co*-EDTM), (**c**) P_8:1_(TT−OMe-*co*-EDTM), and (**d**) P_4:1_(TT−OMe-*co*-EDTM) electrodes based on dual function electrochromic-supercapacitor device.

The energy storage capacity of polymer-based electrochromic supercapacitor devices was assessed, as shown in Figure 9 and Figure S7. The GCD curves of PTT-OMe exhibit its poor electrochemical performance, but there is a clear color change in the process. It can be seen that the working electrode coated on the ITO surface is light yellow in the charging state and purple in the discharging state. With the addition of EDTM, the charging and discharging performances of the copolymer film are improved, which is attributed to increased electronic exchange [59]. This visually recognizable and reversible color change reflects the states of charge and discharge. Moreover, the gradual return to the initial state after charged/discharged indicates good cycle reversibility. The above results indicate that P(TT-OMe-*co*-EDTM) is a promising bifunctional electrode material for electrochromic energy storage.

## 4. Conclusions

In this work, a novel conducting polymer of P(TT-OMe-*co*-EDTM) was synthesized via the one-step electrochemical copolymerization of TT-OMe and EDTM monomers. By optimizing the ratio of TT-OMe to EDTM, copolymer film with a uniform microporous morphology can be fabricated, and the resulting free-standing film exhibits robust mechanical flexibility. Such a P(TT-OMe-*co*-EDTM) film combines the advantageous properties of the two types of conductive polymer materials, endowing it with both an excellent electrochromic performance and electrochemical energy storage capabilities. These copolymer film electrodes displayed enhanced specific capacitance (190 F/g at 5 mV/s) compared to those of the individual PTT-OMe (104 F/g) and PEDTM (119 F/g) electrodes. Furthermore, they demonstrated an excellent electrochromic performance, undergoing significant reversible color changes within a voltage range of −0.4 V to 1.4 V. The P(TT-OMe-*co*-EDTM) polymer films displayed three distinct color changes at different voltages. These reversible color transitions provide visual feedback on energy storage level changes during the charging and discharging processes. This study presents a straightforward and effective approach for fabricating bifunctional conducting polymer electrodes, with promising prospects for future applications in electrochromic energy storage devices.

## Data Availability

Data are contained within the article and Supplementary Materials.

## Supplementary Materials

The following supporting information can be downloaded at: https://www.mdpi.com/article/10.3390/polym16162313/s1, Figure S1. CVs of 0.05 M EDTM in ACN-Bu4NPF6 at 50 mV s^−1^. Figure S2. SEM images of PTT-OMe coated ITO glass. Figure S3. CV curves of 0.05 M (a) PEDTM, (b) PTT-OMe in ACN-Bu4NPF6 at different scan rates, and (c) Plots of specific capacitances versus scan rates of polymers. Figure S4. Cycle stability of PTT-OMe, PEDTM and P8:1(TT-OMe-co-EDTM) electrodes. Figure S5. Charging-discharging of 0.05 M (a) PTT-OMe (b) PEDTM in ACN-Bu4NPF6 at different current densities. (c) Plots of specific capacitances versus current densities of polymers. Figure S6. UV-Vis-NIR spectroscopy, absorption spectra of PEDTM. Figure S7. Charging-discharging curves and corresponding color change of PEDTM based electrochromic-capacitance device. Table S1. EDS analyse of P8:1(TT-OMe-co-EDTM) copolymer film. Table S2. Comparison of the specific capacitance and color change of the materials in this study with other reported similar materials of conducting polymers.
