# Supplementary material for: 3,6-Dimethoxythieno[3,2-b]thiophene-Based Bifunctional Electrodes for High-Performance Electrochromic Supercapacitors Prepared by One-Step Electrodeposition
Source: Polymers (Basel). 2024 Aug 15;16(16):2313. doi: 10.3390/polym16162313 (PMC11359075; doi:10.3390/polym16162313)
Supplement: Supplementary file 1 [file polymers-16-02313-s001.zip › polymers-3147791-supplementary.pdf]

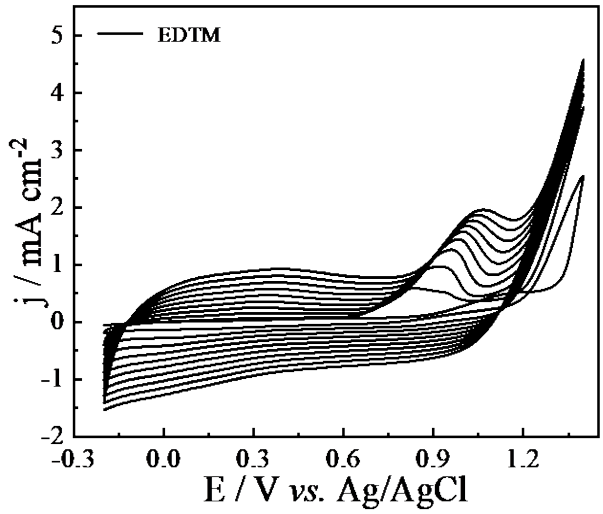

Figure S1 CVs of 0.05 M EDTM in ACN-$Bu4NPF_6$ at 50 mV s^{-1}.

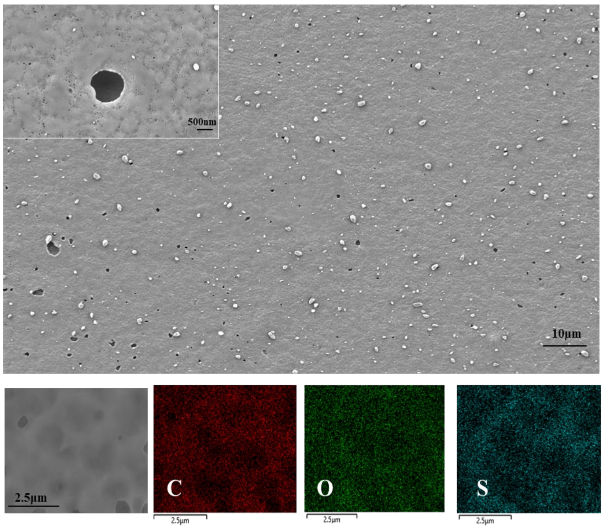

Figure S2 SEM images of PTT-OMe coated ITO glass.

Table S1 EDS analyse of $P_{8:1}$(TT-OMe-*co*-EDTM) copolymer film.

Element	Series	Wt (%)	Wt(σ) (%)	At (%)
C	K-series	41.66	0.52	49.96
O	K-series	52.83	0.50	47.57
S	K-series	5.51	0.15	2.48
Total		100.00		100.00

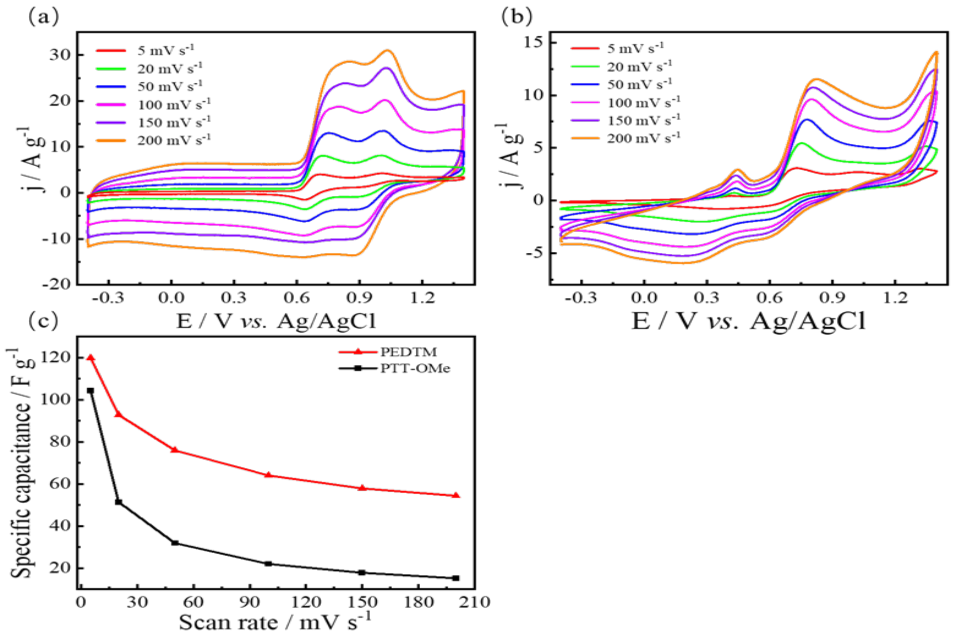

Figure S3 CV curves of 0.05 M (a) PEDTM, (b) PTT-OMe in ACN-Bu_4NPF_6 at different scan rates, and (c) Plots of specific capacitances versus scan rates of polymers.

Table S2 Comparison of the specific capacitance and color change of the materials in this study with other reported similar materials of conducting polymers.

Material	Specific capacitance [F/g]	Color changes	Ref
PPy@$MnMoO_4$	221.3	-	[1]
PANI/PVA	-	Green/Yellow/Blue	[1]
PANI@CNT	207.4	Yellow/ Green/Dark blue	[2]
P(BT-4EDOT)	66.3	Violet/Blue	[3]
PEDOT	171	Blue/Light blue	[4]
$P_{8:1}$(TT-*co*-EDOT)	169	Dark red/orange/light blue	[4]
P3HT/EV/WS2/WO3	50	Magenta/Blue	[5]
P4Fu	246.1	Red/Green/Black	[6]
PFPTT	120	Deeper orange/Tan/Blue	[7]
MoO_3/PPy	129	-	[8]
PEDOT/PPy	290	-	[9]
PTT	94	Orange/Light blue	[10]
PABTS	-	Yellow/ Brown/ greenish blue	[11]
PEDTM	119	Navy blue/Pale blue	This work
PTT-OMe	104	Yellow/Brown/Purple	This work
$P_{8:1}$ (TT-OMe-co-EDTM)	190	Yellow/Brown/Pale purple	This work

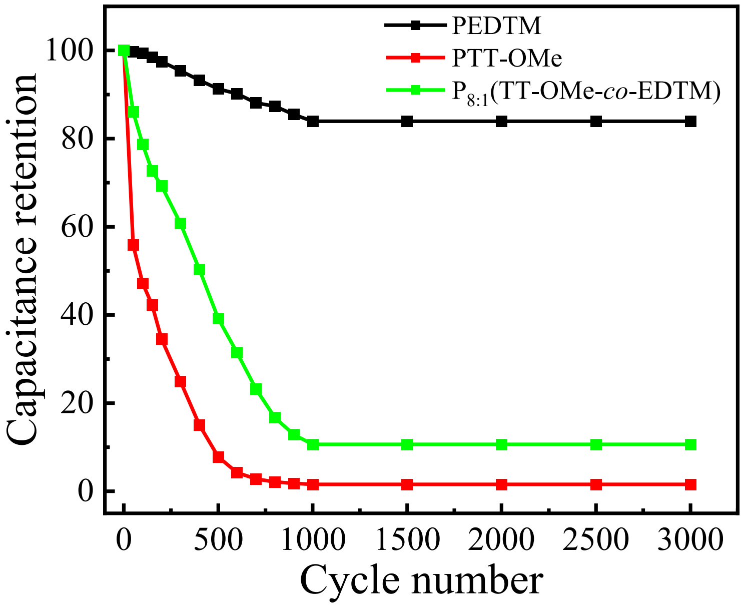

Figure S4 Cycle stability of PTT-OMe, PEDTM and $P_{8:1}$(TT-OMe-*co*-EDTM) electrodes.

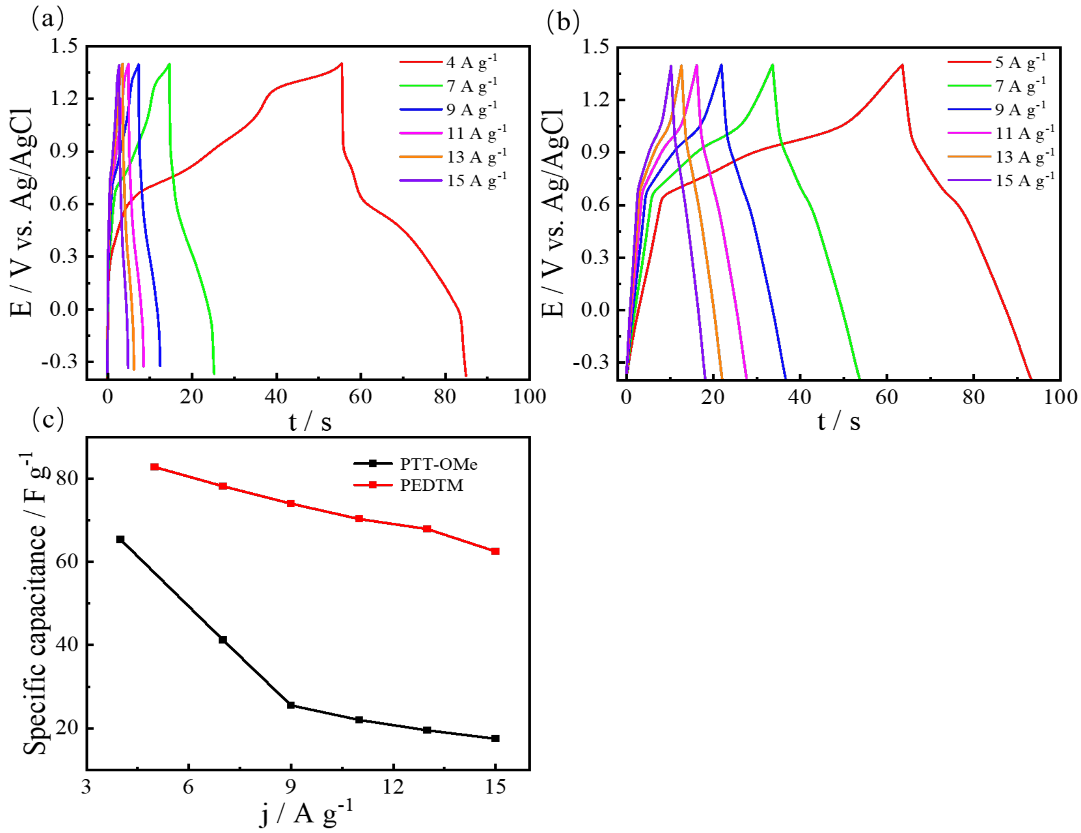

Figure S5 Charging-discharging of 0.05 M (a) PTT-OMe (b) PEDTM in ACN-Bu_4NPF_6 at different current densities. (c) Plots of specific capacitances versus current densities of polymers.

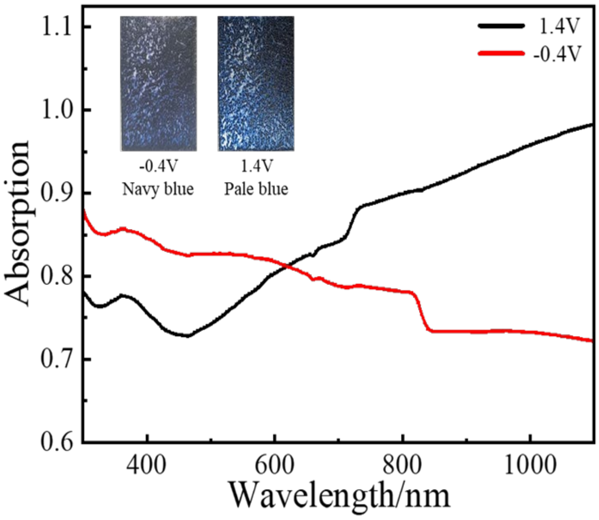

Figure S6 UV-Vis-NIR spectroscopy, absorption spectra of PEDTM.

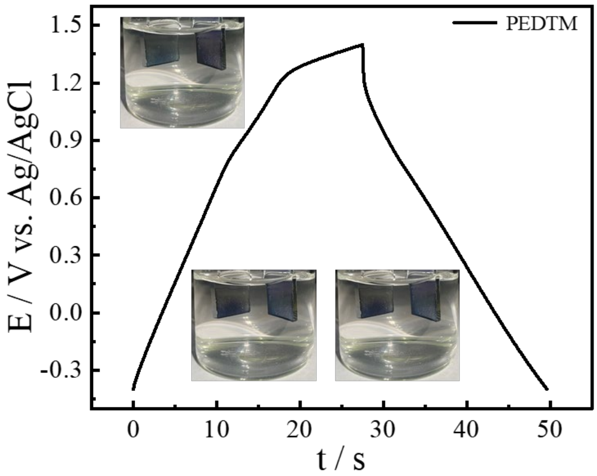

Figure S7 Charging-discharging curves and corresponding color change of PEDTM based electrochromic-capacitance device.

References

[1] D. Mohanadas, Y. Sulaiman, Recent advances in development of electroactive composite materials for electrochromic and supercapacitor applications, Journal of Power Sources 523 (2022) 231029.

[2] K. Xu, Q. Zhang, Z. Hao, Y. Tang, H. Wang, J. Liu, H. Yan, Integrated electrochromic supercapacitors with visual energy levels boosted by coating onto carbon nanotube conductive networks, Solar Energy Materials and Solar Cells 206 (2020) 110330.

[3] H. Li, J. Cao, F. Liu, W. Zhou, X. Chen, Y. Deng, Z. Wu, B. Lu, D. Mo, J. Xu, G. Zhang, Stable Three-Dimensional PEDOT Network Construction for Electrochromic-Supercapacitor Dual Functional Application, ACS Applied Energy Materials 5(10) (2022) 12315-12323.

[4] R. Wang, J. Li, L. Gao, J. Yu, One-step electropolymerized thieno[3,2-b]thiophene-based bifunctional electrode with controlled color conversion for electrochromic energy storage application, Chemical Engineering Journal 445 (2022) 136731.

[5] S. Kandpal, L. Bansal, A. Ghanghass, T. Ghosh, C. Rani, B. Sahu, D.K. Rath, R. Bhatia, I. Sameera, R. Kumar, Bifunctional solid state electrochromic device using WO3/WS2 nanoflakes for charge storage and dual-band color modulation, Journal of Materials Chemistry C 11(37) (2023) 12590-12598.

[6] W. Yao, P. Liu, C. Liu, J. Xu, K. Lin, H. Kang, M. Li, X. Lan, F. Jiang, Flexible conjugated polyfurans for bifunctional electrochromic energy storage application, Chemical Engineering Journal 428 (2022) 131125.

[7] C. Du, H. Li, G. Zhang, R. Wan, W. Zhang, X. Xu, L. Zheng, X. Deng, J. Xu, B. Lu, G. Nie, Design of robust fluorinated interpenetrating poly(thieno[3,2-b]thiophene) network for highly stable flexible electrochromic-supercapacitor devices, Chemical Engineering Journal 495 (2024) 153692.

[8] X. Zhang, X. Zeng, M. Yang, Y. Qi, Investigation of a Branchlike MoO3/Polypyrrole Hybrid with Enhanced Electrochemical Performance Used as an Electrode in Supercapacitors, ACS Applied Materials & Interfaces 6(2) (2014) 1125-1130.

[9] J. Wang, Y. Xu, X. Chen, X. Du, Electrochemical supercapacitor electrode material based on poly(3,4-ethylenedioxythiophene)/polypyrrole composite, Journal of Power Sources 163(2) (2007) 1120-1125.

[10] P.M. Beaujuge, J.R. Reynolds, Color Control in π-Conjugated Organic Polymers for Use in Electrochromic Devices, Chemical Reviews 110(1) (2010) 268-320.

[11] H.-K. Song, E.J. Lee, S.M. Oh, Electrochromism of 2,2′-Azinobis(3-ethylbenzothiazoline-6-sulfonate) Incorporated into Conducting Polymer as a Dopant, Chemistry of Materials 17(9) (2005) 2232-2233.
